# An atypical phosphodiesterase capable of degrading haloalkyl phosphate diesters from *Sphingobium* sp. strain TCM1

**DOI:** 10.1038/s41598-017-03142-9

**Published:** 2017-06-06

**Authors:** Katsumasa Abe, Naoko Mukai, Yuka Morooka, Takeshi Makino, Kenji Oshima, Shouji Takahashi, Yoshio Kera

**Affiliations:** 1 Department of Bioengineering, Nagaoka University of Technology, Nagaoka, Niigata Japan; 2 Department of Biological and Chemical Systems Engineering, National Institute of Technology, Kumamoto College, Yatsushiro, Kumamoto Japan

## Abstract

*Sphingobium* sp. strain TCM1 can degrade tris(2-chloroethyl) phosphate (TCEP) to inorganic phosphate and 2-chloroethanol. A phosphotriesterase (PTE), phosphodiesterase (PDE) and phosphomonoesterase (PME) are believed to be involved in the degradation of TCEP. The PTE and PME that respectively catalyze the first and third steps of TCEP degradation in TCM1 have been identified. However, no information has been reported on a PDE catalyzing the second step. In this study, we identified, purified, and characterized a PDE capable of hydrolyzing haloalkyl phosphate diesters. The final preparation of the enzyme had a specific activity of 29 µmol min^−1^ mg^−1^ with bis(*p*-nitrophenyl) phosphate (B*p*NPP) as the substrate. It also possessed low PME activity with *p*-nitrophenyl phosphate (*p*NPP) as substrate. The catalytic efficiency (*k*_cat_/*K*_m_) with B*p*NPP was significantly higher than that with *p*NPP, indicating that the enzyme prefers the organophosphorus diester to the monoester. The enzyme degraded bis(2,3-dibromopropyl) phosphate, bis(1,3-dichloro-2-propyl) phosphate and bis(2-chloroethyl) phosphate, suggesting that it is involved in the metabolism of haloalkyl organophosphorus triesters. The primary structure of the PDE from TCM1 is distinct from those of typical PDE family members and the enzyme belongs to the polymerase and histidinol phosphatase superfamily.

## Introduction

The chlorinated organophosphorus triesters tris(2-chloroethyl) phosphate (TCEP) and tris(1,3-dichloro-2-propyl) phosphate (TDCPP) are widely used as flame retardants, mainly in a range of plastic foams, resins, and latexes^1^. Their widespread use has led to contamination of various environments, including house dust^2^, surface water^3^, and groundwater^4^. These chemicals are physicochemically and microbiologically stable in the environment and are also reported to be toxic. For example, TCEP was shown to cause adverse effects on various tissues including brain, liver, and kidney^5^, and is also suspected to be carcinogenic^6^. In addition, TDCPP induced genotoxicity in several *in vitro* assays conducted in prokaryotic and eukaryotic cells^7^. These chlorinated organophosphorus triesters are thus a serious threat to human and ecosystem health.

We have previously isolated a TCEP and TDCPP-degrading bacterium, *Sphingobium* sp. strain TCM1. Subsequently, a phosphotriesterase catalyzing the first step of TCEP and TDCPP degradation, named haloalkylphosphorus hydrolase (HAD), was cloned and purified^8, 9^. We also recently reported that two alkaline phosphatases, which are metal-dependent phosphomonoesterases (PMEs) named SbPhoA and SbPhoX-II, are involved in the metabolism of TCEP^10^. However, no information has been reported on a phosphodiesterase (PDE) catalyzing the second step of TCEP degradation. Since the previous studies in our laboratory indicated that the rate-limiting step in the degradation pathway of these triesters in strain TCM1 is the step catalyzed by PDE or PME, identification of the PDE is important to improve the biodegradation of haloalkyl phosphorus compounds using TCM1.

PDEs can degrade the phosphodiester bond of various compounds, and include cyclic nucleotide PDEs, phospholipase C, phospholipase D and some nucleases. Cyclic nucleotide PDEs are among the most characterized PDEs; they hydrolyze the phosphodiester bond of cyclic nucleotides and play a critical role in intracellular signaling by selectively hydrolyzing the second messengers cAMP and cGMP^11^. The cyclic nucleotide PDEs can be classified into three different groups by their primary structure. Class I PDEs are found exclusively in higher eukaryotes and are further classified into 11 families^11^. Class II PDEs have been identified in a few organisms, such as yeast, *Dictyostelium* and *Vibrio fischeri* ^12, 13^. All class III PDEs are found in prokaryotes, including *Escherichia coli* ^14^, *Mycobacterium tuberculosis* ^15^ and *Arthrobacter* ^16^. Only a few studies have focused on the biodegradation of toxic organophosphate compounds by PDEs. As an example, the glycerophosphodiester phosphodiesterase (GpdQ) from *Enterobacter aerogenes* is one of the few can hydrolyze a broad range of phosphodiesters, including dimethyl phosphate, diethyl phosphate, bis(*p*-nitrophenyl) phosphate (B*p*NPP), cAMP and EA 2192, a degradation product of the nerve agent VX^17–19^.

In the present paper, we describe the identification, purification and characterization of the PDE from *Sphingobium* sp. TCM1, which is named *Sb*-PDE. This enzyme hydrolyzes dihaloalkyl phosphates such as bis(2-chloroethyl) phosphate (BCEP), bis(1,3-dichloro-2-propyl) phosphate (BDCPP) and bis(2,3-dibromopropyl) phosphate (BDBPP), which are hydrolysis products of the corresponding trihaloalkyl phosphates, TCEP, TDCPP and tris(2,3-dibromopropyl) phosphate (TDBPP). The primary structure of *Sb*-PDE is distinct from those of typical PDE family members and the enzyme belongs to the polymerase and histidinol phosphatase (PHP) superfamily.

## Results

### Purification of *Sb*-PDE from *Sphingobium* sp. TCM1

**Figure 1:**
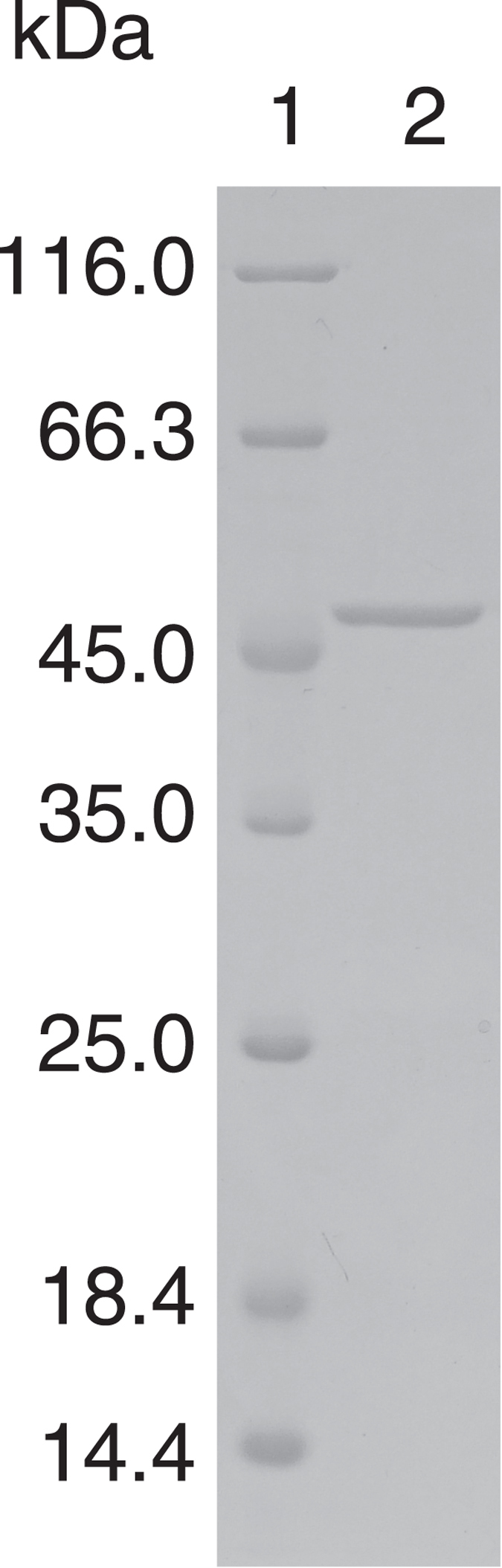
SDS-polyacrylamide gel electrophoresis of purified *Sb*-PDE from *Sphingobium* sp. TCM1. Protein samples were separated on a 12.5% SDS-polyacrylamide gel and stained with Coomassie Brilliant Blue R-250. Lane 1, marker proteins; lane 2, purified enzyme (1 µg of protein).

*Sb*-PDE was purified to homogeneity from *Sphingobium* sp. TCM1 by chromatography using phenyl Sepharose HP, Q Sepharose HP and Superdex 200 pg columns, with a yield of 14.8% (Table 1). The specific activity toward B*p*NPP was 29.4 µmol min^−1^ mg^−1^ and the final purification was 147-fold. The purified enzyme migrated as a single band in SDS-PAGE with an apparent molecular mass of 48.6 kDa (Fig. 1), and the molecular mass of the native enzyme was found to be 116 kDa by gel filtration with Superdex 200 pg. These results suggest that the enzyme consists of a dimer of identical subunits.

**Table 1:** Purification of PDE from *Sphingobium* sp. TCM1^a^.

Step	Total activity^b^ (µmol · min^−1^)	Total protein (mg)	Specific activity (µmol · min^−1^ · mg^−1^)	Purification (fold)	Yield (%)
Crude extract	463	2312	0.20	1.00	100
Ammonium sulfate fractionation	366	491	0.74	3.72	79.0
Phenyl Sepharose HP	251	202	1.24	6.19	54.2
Q Sepharose HP	94.9	5.35	17.7	88.6	20.5
Superdex 200 pg	68.4	2.33	29.4	147	14.8

^a^Starting material was 47.4 g of wet cells.

^b^The activity of *Sb*-PDE was measured using the standard assay mixture with B*p*Npp as the substrate.

PME activity of the enzyme was also determined during purification (Supplementary Table S1). The purified enzyme had PME activity and the ratio of PDE activity to PME activity was 3.9.

### Effects of pH and temperature on enzyme activity

**Figure 2:**
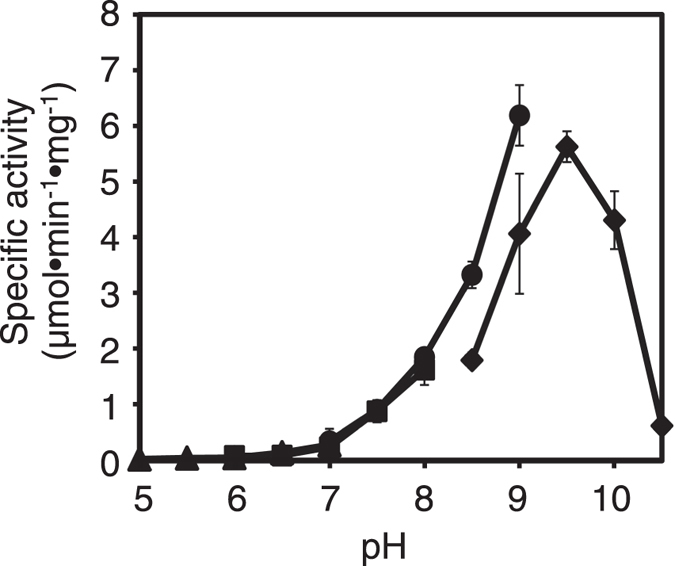
Effect of pH on PDE activity. Enzyme activity was measured in 50 mM MES buffer (closed triangles), 50 mM MOPS buffer (closed squares), 50 mM Tris-HCl buffer (closed circles) or 50 mM glycine-NaOH buffer (closed diamonds). The final concentration of purified *Sb*-PDE in the assay mixture was 0.17 µg/ml. The data are means ± standard deviations from three independent experiments.

To determine the optimal pH of the enzyme, its activity was determined in the standard assay conditions at 30 °C using B*p*Npp as the substrate at pH 5.0–10.5 (Fig. 2). The enzyme showed maximum activity at pH 9.5 in 50 mM Glycine-NaOH buffer and high activity at pHs in the range 9.0–10.5.

**Figure 3:**
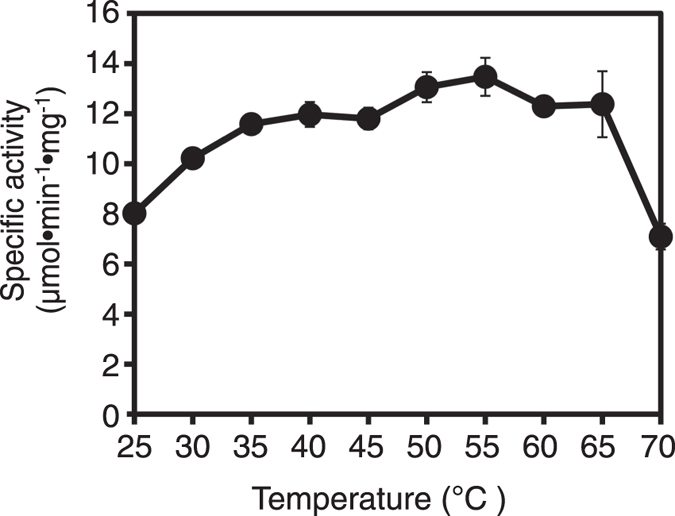
Effect of temperature on PDE activity. The activity of *Sb*-PDE was measured using the standard assay mixture with B*p*Npp as the substrate at 25–70 °C. The final concentration of purified *Sb*-PDE in the assay mixture was 0.07 µg/ml. The data are means ± standard deviations from three independent experiments.

The effect of temperature on the enzyme activity was also determined using the standard assay mixture and B*p*Npp as the substrate (Fig. 3). The optimal temperature for activity was 55 °C and the enzyme showed relatively high activity in the temperature range 45–65 °C.

### Kinetic parameters

Kinetic parameters of the enzyme were determined using 0.25–20 mM B*p*NPP or *p*Npp as substrate, respectively. The *K*_m_ an*d k*_cat_ values with B*p*NPP were 6.1 ± 1.2 mM and 325 ± 46 s^−1^, respectively. The enzyme did not follow Michaelis–Menten kinetics because it is subject to substrate inhibition at high B*p*NPP concentrations (Supplementary Figure S1); the *K*_i_ value was 12.5 ± 3.1 mM. However, the enzyme followed Michaelis-Menten kinetics with *p*NPP, and the *K*_m_ an*d k*_cat_ values were 1.5 ± 0.2 mM and 37.9 ± 2.4 s^−1^, respectively.

### Effects of various compounds on enzyme activity and metal ion analysis

The effects of various metal salts, chelating agents, and thiol-modifying reagents on the PDE activity were examined (Table 2). *Sb*-PDE was moderately activated by the addition of Mn^2+^ or Zn^2+^, but other divalent metal ions did not affect the enzyme. EDTA and EGTA had no effect on the activity, while the enzyme activity was largely inhibited by *o*-phenanthroline. Dithiothreitol or 2-mercaptoethanol significantly inhibited the activity of *Sb*-PDE, whereas *N*-ethylmaleimide and *p*-chloromercuribenzoic acid did not.

**Table 2:** Effects of various compounds and metal ions on the activity of *Sb*-PDE from *Sphingobium* sp. TCM1.

Compound	Concentration (mM)	Relative activity (%)
None	—	100
MgCl_2_	0.1	100
CaCl_2_	0.1	105
MnCl_2_	0.1	131
FeCl_2_	0.1	96
CoCl_2_	0.1	95
NiCl_2_	0.1	101
CuCl_2_	0.1	87
ZnCl_2_	0.1	128
EDTA	1	95
	10	110
EGTA	1	98
	10	105
*o*-Phenanthroline	1	2
Dithiothreitol	1	4
2-Mercaptoethanol	1	46
*N*-Ethylmaleimide	1	98
*p*-Chloromercuribenzoic acid	1	107

The activity of *Sb*-PDE was measured using the standard assay mixture with B*p*Npp as the substrate. The final enzyme concentration was 1.9 µg/ml. The activity without addition of metal ions or compounds was defined as 100%.

ICP-MS analysis of *Sb*-PDE showed that 2.1 equivalent of zinc and 0.05 equivalent of cobalt bound to the enzyme, whereas other metals including iron, calcium, nickel, manganese and magnesium were not detected.

### Substrate specificity

The substrate specificity of *Sb*-PDE toward BCEP and BDCPP was examined in stepwise reactions with haloalkylphosphorus hydrolase (see Methods) because these compounds were not available from commercial sources. *Sb*-PDE could hydrolyze the bis-haloalkyl phosphates BCEP, BDCPP and BDBPP with specific activities of 0.007, 2.02 and 19.5 µmol min^−1^ mg^−1^, respectively. The enzyme also displayed PDE activities toward cAMP and cGMP (0.017 and 0.008 µmol min^−1^ mg^−1^, respectively).

### Identification of the *Sb*-*pde* gene and expression in *E*. *coli*

The N-terminal amino acid residues of *Sb*-PDE purified from *Sphingobium* sp. strain TCM1 were determined to be EFVTQSDQRPP. We performed a Blast search against the draft genome of strain TCM1^20^ using this N-terminal sequence as the query sequence and the putative gene (locus_tag A7Q26_24300) was found. However, no Shine-Dalgarno (SD) sequence was found around the putative translation initiation codon. Moreover, the N-terminal region of the deduced amino acid sequence from the putative *Sb*-*pde* gene was 15 residues shorter than that of a hypothetical protein from *Novosphingobium resinovorum* (accession no. WP_006949123), which otherwise showed 100% amino acid identity with the deduced amino acid sequence of *Sb*-PDE. We found another initiation codon 45 bp upstream of the previous candidate start codon of the predicted gene (corresponding to 15 amino acid residues), with a SD sequence (5′-aggaga-3′) 4-bp upstream of the codon. From these results, we concluded that the newly found initiation codon could function as the translation initiation site. The putative *Sb*-*pde* gene (accession no. LC177422) contained an open reading frame of 1,326 nucleotides encoding a protein of 441 amino acid residues with a predicted molecular weight of 48,702.

The N-terminal amino acid sequence of the purified enzyme was identical to amino acid residues 29–39 of the putative amino acid sequence of *Sb*-PDE predicted from the gene. An N-terminal signal peptide was predicted by SignalP analysis and the putative cleavage site is consistent with the result of N-terminal amino acid sequence analysis of purified *Sb*-PDE. Thus, *Sb*-PDE contains a signal peptide for secretion, and the mature protein consists of 413 amino acids with calculated molecular weight 45,769.

**Figure 4:**
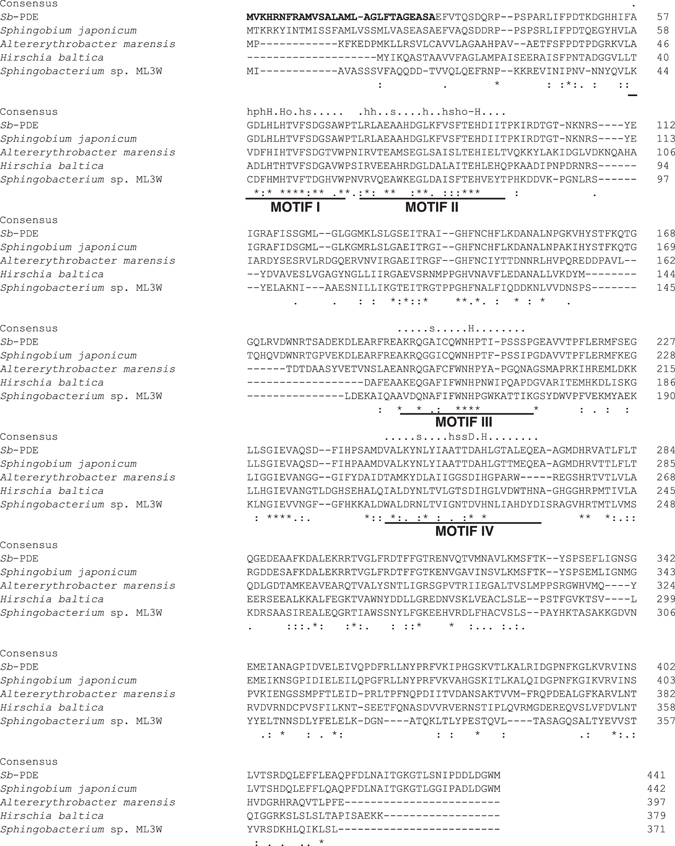
Sequence alignment of *Sb*-PDE with hypothetical proteins from *Sphingobium japonicum* (accession no. WP_006949123), *Altererythrobacter marensis* (WP_047806715), *Hirschia baltica* (WP_015828317) and *Sphingobacterium* sp. ML3W (WP_051959780). The numbers on the right are the residue numbers for each amino acid sequence. Identical residues and amino acid substitutions with low and high similarities are indicated by asterisks, dots and double dots, respectively. The putative signal peptide of *Sb*-PDE is in bold. The four conserved motifs in the PHP domain are underlined. The consensus sequence of each motif^24^ is shown above the alignment; h indicates hydrophobic residues (A, C, F, I, L, M, V, W or Y), s indicates small residues (A, C, S, T, D, N, V, G or P), p indicates polar residues (D, E, H, K, N, Q, R, S or T), o indicates hydroxy residues (S or T), and ‘−’ indicates negatively charged residues (D or E).

The deduced amino acid sequence of the enzyme showed 100% identity with a hypothetical protein from *N*. *resinovorum* (accession no. EZP79922) and significant similarity with a hypothetical protein from *S*. *japonicum* (85% identity) (Fig. 4). Although the functions of these hypothetical proteins are unknown, both proteins are members of the PHP superfamily. Motif analysis of *Sb*-PDE using the MOTIF program (GenomeNet, http://www.genome.jp/tools/motif/) also revealed that *Sb*-PDE contains the PHP domain (Pfam accession number PF02811), which is found in bacterial polymerase III α subunits, family X DNA polymerases and histidinol phosphate phosphatases (Fig. 4).

The *Sb*-*pde* gene containing the putative signal peptide coding region of strain TCM1 was expressed in *E*. *coli* BL21 (DE3) cells under the control of the T7 promoter. Crude extracts of *E*. *coli* cells harboring pET25b showed neither PDE nor PME activity, while those of *E*. *coli* cells harboring pET25b-*pde* showed hydrolase activities of 7.01 µmol min^−1^ mg^−1^ for B*p*Npp and 1.97 µmol min^−1^ mg^−1^ for *p*Npp (PDE activity/PME activity = 3.6), indicating that the expressed gene encodes *Sb*-PDE. The N-terminal amino acid sequence of recombinant *Sb*-PDE, EFVTQXDQ (X represents an unidentified amino acid residue), was identical with that of native mature enzyme suggesting that the recombinant *Sb*-PDE was also processed in *E*. *coli*.

## Discussion

*Sphingobium* sp. strain TCM1, which was isolated previously in our laboratory from soil^8^, can degrade TCEP to inorganic phosphate and 2-chroloethanol. It is considered that a phosphotriesterase, PDE and PME are involved in the degradation of TCEP. While phosphoesterases catalyzing the first and third steps of TCEP degradation in TCM1 have been identified in our previous studies as a HAD^9^ and an alkaline phosphatase^10^ respectively, no information has been reported on a PDE from this microorganism. In the present study, *Sb*-PDE from *Sphingobium* sp. TCM1 was purified and characterized and the gene was cloned.

BDCPP and BDBPP were efficiently degraded by *Sb*-PDE, whereas the enzyme showed only weak activity toward BCEP, cAMP and cGMP. TCM1 can degrade both TCEP and TDCPP, but the generation of 2-CE from TCEP was slower than that of 1,3-DCP from TDCPP^8^. BCEP may be a relatively poor substrate for *Sb*-PDE. These results suggest a possible involvement of *Sb*-PDE in the metabolism of haloalkyl phosphates in TCM1, although involvement of the enzyme in cyclic nucleotide(s) metabolism was not established.

The enzyme contained a predicted signal peptide for secretion and the mature protein is a homodimer with a subunit molecular mass of 45.8 kDa. No consensus twin-arginine-transport (TAT) motif is found in this signal peptide, suggesting that the enzyme is probably secreted in a Sec-dependent manner. *Sb*-PDE was inhibited by dithiothreitol and 2-mercaptoethanol. Since *Sb*-PDE is possibly secreted extracellularly and has two cysteine residues, disulfide bond essential for enzyme activity or protein folding may be present in the enzyme.

It was reported that PDEs such as GpdQ from *Enterobacter aerogenes* ^19^ and PdeA from *Delftia acidovorans* ^21^ exhibit PME activity. Furthermore, alkaline phosphomonoesterases, such as PhoA from *Escherichia coli* ^22^ and PhoD from *Aphanothece halophytica* ^23^, exhibit both PME and PDE activity. Therefore, we determined the PME activity of *Sb*-PDE. The purified enzyme had PME activity in addition to PDE activity, but the PDE activity was 3.9-fold lower. Kinetic analysis revealed that the catalytic efficiency (*k*_cat_ */K*_m_) with B*p*NPP was significantly higher than that with pNPP. These results indicate that this enzyme is a PDE.

*Sb*-PDE hydrolyzed both a phosphodiester and a phosphomonoester. However, it has already been reported that two alkaline phosphatases, SbPhoA and SbPhoX-II, are involved in the utilization of TCEP by *Sphingobium* sp. strain TCM1 and the double gene mutant did not grow on TCEP medium^10^. These results suggest that *Sb*-PDE catalyzes only the second step in the degradation of organophosphorus triesters, and not the third (phosphomonoester hydrolysis) step, although the PME activity of the enzyme toward monohaloalkyl phosphate was not determined in this study.

The primary structure of *Sb*-PDE from TCM1 is distinct from those of typical PDE family members. *Sb*-PDE contained a PHP domain, which is part of the amidohydrolase superfamily, while no consensus sequences of class I, class II or class III PDEs were found. The PHP domain is found in bacterial polymerase III α subunits, family X DNA polymerases and histidinol phosphate phosphatases and the biochemical functions of this domain in these enzymes are well characterized. The C-terminal PHP domain of DNA polymerase X shows Mn^2+^-dependent 3′, 5′-exonuclease activity^24, 25^ and histidinol phosphatase catalyzes the dephosphorylation of l-histidinol phosphate to histidinol^26^. The PHP superfamily also includes numerous proteins of unknown function^24^. In fact, many proteins with sequence similarity to *Sb*-PDE are annotated as hypothetical proteins in databases. Although it was reported that Elen0235 from *Eggerthella lenta* can hydrolyze a cyclic phosphodiester to a vicinal diol and inorganic phosphate^27^, little is known about whether proteins belonging to the PHP superfamily display PDE activity.

The PHP motif is predicted to be involved in metal-dependent phosphoester bond hydrolysis^24^, and structurally characterized enzymes of the PHP superfamily contain a trinuclear metal center in the active site^28–31^. *Sb*-PDE and several homologous proteins have the four conserved motifs^24^ that have been predicted to be involved in the formation of the metal binding sites (Fig. 4). Furthermore, the crystal structure analysis of l-histidinol phosphate phosphatase from *Lactococcus lactis* subsp. *lactis* Il1403 showed that nine amino acid residues are metal coordination residues in the active site of the enzyme^31^. These amino acid residues are highly conserved in l-histidinol phosphate phosphatases from various microorganisms, and also in *Sb*-PDE (Supplementary Figure S2). In addition, the activity of purified *Sb*-PDE was markedly inhibited by the addition of *o*-phenanthroline (Table 2), and ICP-MS analysis revealed that the enzyme contains zinc (more than 97% of total metals) and cobalt. These observations suggest that the enzyme mainly binds zinc ions as a cofactor.

Previous studies in our laboratory indicated that the rate-limiting step in the degradation pathway of the triesters in strain TCM1 is probably the step catalyzed by PDE or PME. The present study provides valuable information not only for understanding the function of a hypothetical protein belonging to the PHP superfamily but also for development of haloalkyl-phosphate-degrading microbial systems.

## Methods

### Materials

BDBPP and TDCPP were purchased from Wako Pure Chemical Industries (Osaka, Japan). B*p*NPP and TCEP were from Tokyo Kasei Kogyo Co. Ltd. (Tokyo, Japan). *p*-Nitrophenyl phosphate sodium salt (*p*NPP) was from Nacalai Tesque (Kyoto, Japan). Restriction enzymes and other DNA-modifying enzymes were obtained from Takara Shuzo (Kyoto), Toyobo (Osaka) and New England Biolabs (Beverly, MA). DNA purification kits were from Qiagen (Valencia, CA). Phenyl Sepharose HP, Q Sepharose HP and Superdex 200 pg columns were from GE Healthcare UK Ltd. (Buckinghamshire, UK). All other chemicals were of analytical purity and were purchased from Wako Pure Chemical Industries and Nacalai Tesque. The LC-10Ai Bio-Inert high-performance liquid chromatography (HPLC) system was from Shimadzu (Kyoto).

### Bacterial strain and culture condition

*E*. *coli* BL21 (DE3) was used as the host for the expression of *Sb*-PDE. *Sphingobium* sp. TCM1 was aerobically cultivated in MAY medium containing 20 µM TCEP as the sole phosphorus source^32^. *E*. *coli* BL21 (DE3) were grown in Luria-Bertani (LB) medium containing 100 µg/ml ampicillin.

### Purification of *Sb*-PDE from *Sphingobium* sp. TCM1

*Sphingobium* sp. TCM1 was cultivated aerobically at 30 °C for 48–50 h and harvested by centrifugation (5,000 × *g* for 10 min). After washing cells twice with 50 mM MOPS buffer (pH 7.4), the cells were resuspended in the same buffer and homogenized by sonication, followed by a centrifugation at 20,000 × *g* for 30 min. The cell free extract thus obtained was fractionated by ammonium sulfate precipitation (30–60% saturation). The resulting precipitate was dissolved in M/G buffer (50 mM MOPS (pH 7.4) containing 10% (v/v) glycerol) containing ammonium sulfate at 30% saturation and loaded onto a phenyl Sepharose HP column (2.6 × 11 cm) equilibrated with same buffer. After the column was washed with M/G buffer containing ammonium sulfate at 30% saturation, the enzyme was eluted by using a linear gradient (540 ml) from 30% to 0% ammonium sulfate at a flow rate of 0.7 ml/min. The fractions with PDE activity were collected, combined and concentrated by using an Amicon stirred cell apparatus with a YM-10 membrane (Millipore, Billerica, MA). After buffer exchange into 50 mM Tris-HCl (pH 8.0) containing 10% (v/v) glycerol (T/G buffer), the concentrate was subjected to a Q Sepharose HP column (2.6 × 11 cm) pre-equilibrated with T/G buffer and the *Sb*-PDE was then eluted by using a linear gradient (360 ml) from 0 to 600 mM NaCl in the T/G buffer at a flow rate of 1.0 ml/min. The active fractions were collected, concentrated and loaded onto a Superdex 200 pg column (1.6 × 60 cm) equilibrated with M/G buffer containing 150 mM NaCl. The enzyme was eluted with the same buffer at a flow rate of 0.5 ml/min and the fractions with the enzyme activity were pooled, concentrated and stored at −80 °C until use.

### Enzyme assays

PDE and PME activities were respectively assayed at 30 °C by monitoring spectrophotometrically the liberation of *p*-nitrophenol from B*p*Npp and *p*Npp. A 1.5-ml standard assay mixture contained 50 mM Tris-HCl buffer (pH 8.8), 15 mM B*p*Npp for PDE assay or 15 mM *p*NPP for PME assay, and an appropriate amount of the enzyme, which was added to the mixture to start the assay. A mixture without enzyme was used as the blank. The activity of the enzyme was determined as the increase in absorbance at 410 nm using a UV-2500PC spectrophotometer (Shimadzu). The kinetic parameters of the enzymes for B*p*NPP and *p*NPP were estimated by fitting the initial reaction rates to the Michaelis-Menten equation with the program SigmaPlot 12.5 (Systat Software, San José, CA). Data are expressed as mean ± S.E. of the three independent experiments.

### Substrate specificity

The hydrolytic activities of *Sb*-PDE toward BCEP, BDCPP and BDBPP were determined by the increase in the amount of 2-chloroethanol, 1,3-dichloro-2-propanol and 2,3-dibromopropanol, respectively, as described below. BCEP was produced by an incubation of TCEP (at the final concentration of 45 μM) with a recombinant His-tagged TCM1-HAD (0.87 μg/ml) with specific activity of 1.86 µmol min^−1^ mg^−1^ (K. Abe, S. Takahashi and Y. Kera, unpublished data) in 50 mM Tris-HCl buffer (pH 8.0) at 30 °C for 24 h. BDCPP was produced from TDCPP in the same way. Complete hydrolysis of TCEP and TDCPP by His-tagged HAD were analyzed by gas chromatography (GC), and the stoichiometric productions of the corresponding halo alcohols were also confirmed by analyses with a gas chromatograph mass spectrometer (GCMS).

To assay PDE activity toward BCEP, 50 µl *Sb*-PDE (570 μg/ml) was added to 1.95 ml of the hydrolytic mixture of TCEP described above, followed by incubation at 30 °C. A mixture without enzyme was used as the blank. An aliquot (200 μl) of the incubation mixture was removed after 0, 15, 30, 60, 90, 120 and 180 min. 2-Chloroethanol in the aliquot was extracted with 1 ml of ethyl acetate and analyzed by GC-MS.

PDE activity toward BDCPP was measured in the same way, but 50 µl of a much lower concentration of *Sb*-PDE (2.85 μg/ml) was added to 1.95 ml of the hydrolytic mixture of TDCPP. An aliquot (200 μl) of the incubation mixture was removed after 0, 15, 30 and 60 min. 1,3-Dichloro-2-propanol in the aliquot was extracted with 1 ml of ethyl acetate and analyzed by GC-MS.

The assay of PDE activity toward BDBPP was carried out at 30 °C, using a mixture (2 ml) containing 50 mM Tris-HCl buffer (pH 8.0), 66 μM BDBPP and 50 µl of *Sb*-PDE enzyme (2.85 μg/ml). An aliquot (200 μl) of the reaction mixture was removed after 0, 15 and 30 min. 2,3-Dibromopropanol in the aliquot was extracted with 1 ml of ethyl acetate and analyzed by GC-MS.

PDE activity toward cAMP and cGMP was measured using a colorimetric cyclic nucleotide phosphodiesterase assay kit (Enzo Life Sciences, Inc. Farmingdale, NY).

### GC and GC-MS analyses

Concentrations of TCEP and TDCPP were analyzed using a GC-17A equipped with a flame photometric detector with a phosphorus optical filter (Shimadzu), as described in a previous report^8^. Concentrations of 2-chloroethanol, 1,3-dichloro-2-propanol and 2,3-dibromopropyl phosphate were determined using a GCMS-QP2010 (Shimadzu), as described^8^.

### Molecular mass determination

The native molecular mass of the enzyme was analyzed by gel filtration with a HPLC equipped with the Superdex 200 pg column. The gel filtration chromatography was performed at a flow rate of 0.5 ml/min with M/G buffer containing 150 mM NaCl. The molecular mass marker proteins (GE Healthcare) consisting thyroglobulin (669 kDa), ferritin (440 kDa), catalase (232 kDa), aldolase (158 kDa), albumin (67 kDa), ovalbumin (43 kDa), chymotrypsinogen A (25 kDa), and ribonuclease A (13.7 kDa) were used as the standard.

### Inductively coupled plasma mass spectrometry

Metal ion content was determined by inductively coupled plasma mass spectrometry using an Agilent7700x (Agilent Technologies, Santa Clara, CA). The purified enzyme (10 nmol) was digested in 5% nitric acid for 24 h to release the metal ions. The sample was then diluted with 5 volume of ultrapure water and was directly subjected to ICP-MS measurement.

### N-terminal amino acid sequencing

Purified enzyme or crude extract of *E*. *coli* cells harboring pET25b-*pde* was resolved by SDS-PAGE. The resolved proteins were transferred to a Sequi-Blot polyvinylidene difluoride (PVDF) membrane (Bio-Rad, Hercules, CA) by electroblotting and the PVDF membrane stained by Coomassie Brilliant Blue R-250. The protein bands on the PVDF membrane were cut out from the membrane, and the N-terminal amino acid sequence of the protein was determined by automated Edman degradation with a Shimadzu model PPSQ-21 protein sequencer.

### Expression of *Sb*-PDE in *E*. *coli*

The *Sb*-*pde* gene was amplified from genomic DNA of *Sphingobium* sp. TCM1 by PCR with primers TCM Pde Ex F1 (5′-AAGGAGATATACATATGGTTAAGCATAGAAACTTCCGCGC-3′) and TCM Pde Ex R1 (5′-AGCAGGTATTTCATATTACATCCAGCCGTCGAGATCG-3′). The resulting PCR product was ligated into *Nde*I-digested pET25b using the In-Fusion HD Cloning Kit (Clontech, Palo Alto, CA). The plasmid for the expression of *Sb*-PDE was named pET25b-*pde*. The pET25b-*pde* was introduced into *E*. *coli* BL21 (DE3), and the cells were grown in LB medium containing 100 µg/ml ampicillin at 30 °C for 3 h. The culture was supplemented with 1 mM isopropyl-1-thio-β-d-galactopyranoside and grown for further 14 h at 20 °C. The cells were harvested, resuspended in 50 mM MOPS buffer (pH 7.4) and homogenized by sonication.

### Other analytical methods

Protein concentration was determined by the Bradford method^33^ using a protein assay kit (Bio-Rad) with bovine serum albumin as the standard. SDS-PAGE in reducing conditions was performed as described by Laemmli^34^.

## Electronic supplementary material

Supplementary information
