# Supplementary material for: An atypical phosphodiesterase capable of degrading haloalkyl phosphate diesters from Sphingobium sp. strain TCM1
Source: Sci Rep. 2017 Jun 6;7:2842. doi: 10.1038/s41598-017-03142-9 (PMC5460133; doi:10.1038/s41598-017-03142-9)
Supplement: Supplementary file 1 — Supplementary information [file 41598_2017_3142_MOESM1_ESM.pdf]

Supplementary Information

An atypical phosphodiesterase capable of degrading haloalkyl phosphate diesters from *Sphingobium* sp. strain TCM1

Katsumasa Abe[1], Naoko Mukai[1], Yuka Morooka[1], Takeshi Makino[1], Kenji Oshima[2], Shouji Takahashi[1], Yoshio Kera[1,*]

[1]Department of Bioengineering, Nagaoka University of Technology, Nagaoka, Niigata, Japan

[2]Department of Biological and Chemical Systems Engineering, National Institute of Technology, Kumamoto College, Yatsushiro, Kumamoto, Japan

*Address correspondence to Yoshio Kera, yoshkera@vos.nagaokaut.ac.jp

Table S1 PDE and PME activities during purification step

Step	Total activity (μmol•min^{-1})		B*p*NPP/*p*NPP
	B*p*NPP	*p*NPP	
Cell-free extract	463	1253	0.4
Ammonium sulfate fractionation	366	1007	0.4
Phenyl Sepharose HP	251	79.0	3.2
Q Sepharose HP	94.9	29.7	3.2
Superdex 200 pg	68.4	17.4	3.9

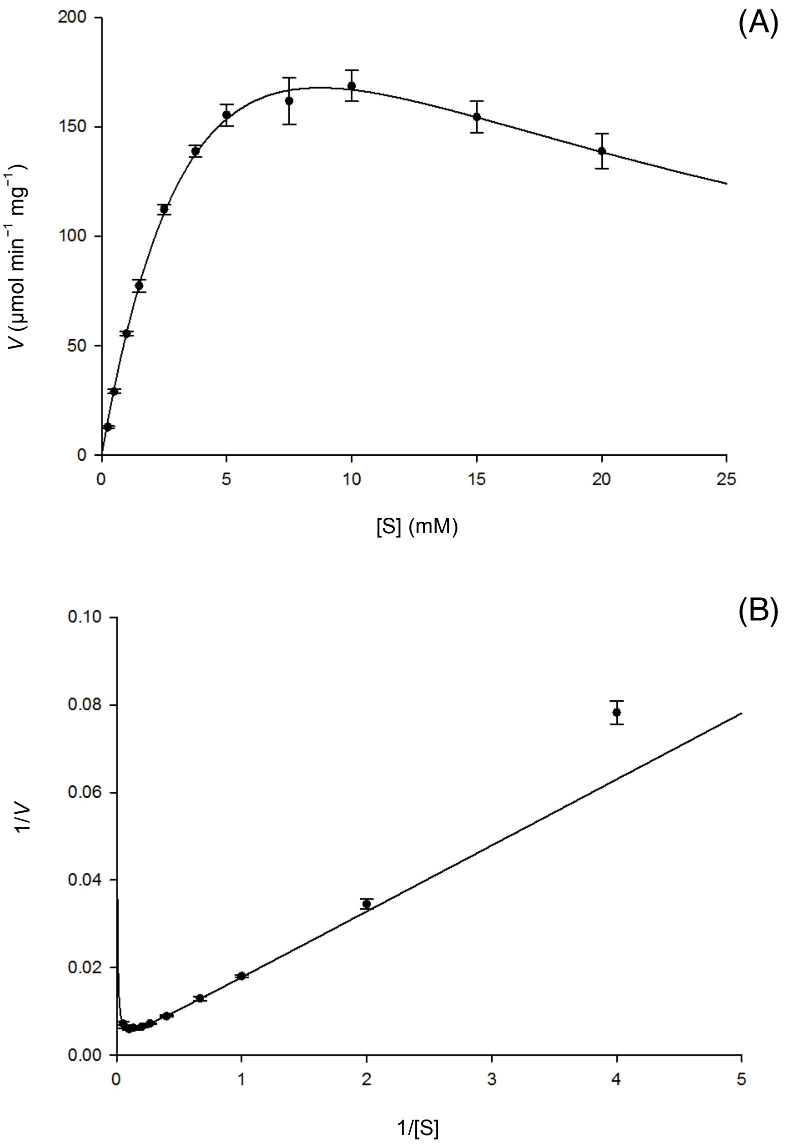

Figure S1 Effect of substrate concentration on the PDE activity (A) and Lineweaver-Burk plot (B) of PDE activity. *V* and [S] indicate velocity and substrate concentration, respectively. The data are means ± standard error (SE) from three independent experiments

```
                       ▼ ▼     ▼
Lla-HPP   1    MSLKKLDYHFHSHFSADSEELP--------------------------------------
Sb-PDE    55   --IFAGDLHLHTVFSDGSAWPT--------------------------------------
Bce-HPP   1    ---MKVDYHIHLEEGPYSIGWLAKINESLQHYEPLKEEKHSMEWLVKIQERLQRRVNEGP
Bwe-HPP   1    ---MKVDYHIHLEEGPYSIGWLAKINDALQYFEPLKEEKHSMEWLMKTQERLQRRVKEGP
Mca-HPP   1    ---MKVDFHIHLEEGPYTNNFFNKTITSIDTVKGIQAT-HTLDDIERKAQLFNERMEKGD
Tth-HPP   1    ----MVDSHVHTPLCGHAEGHP--------------------------------------
Bsu-HPP   1    --MQKRDGHIHTPFCPHGSNDT--------------------------------------
                     * *.*

                                        ▼
Lla-HPP   23   ------RKHVTEAIAHGLEEICFTEHRD-------------------------FYFPGMD
Sb-PDE    75   -------LRLAEAAHDGLKFVSFTEHDIIT----------------------PKIRDTGT
Bce-HPP   58   FTTKWIDLYLEEALRKGIKEVGIVDHLYRFYEAKEYYEKYVDISDSGLGRLQKEWLDQVR
Bwe-HPP   58   FTAKWIDLYLEEAVRKGIKEVGIVDHLYRFHEAKGYYEKYVDISDSRLGRLQKEWLDQVR
Mca-HPP   57   YSEWWLDLYLEMSLQKGLKQVGIVDHLYRFQETRNYFLKYMDVSDTDLGCRQREWLNQVM
Tth-HPP   19   ------EAYLEEARAKGLKGVVFTDHSP----------------------MPPWYDPESR
Bsu-HPP   21   -----LRQYAEEALKKGFESITFTEHAP----------------LPPSFTDPTPLKDSAM
                           :  .*:: : :.:*

                                            ▼                             ▼
Lla-HPP   52   FSLNLPEYFQEINQLQAEFKDKIKIKIGLEMGIDLRFKSEINQFIDSA--PFDFVIASVH
Sb-PDE    106  NKNRSYEIGRAFISSGMLGLGGMKLSLGSEITRAIGHFNCHFLKDANA-----LNPGKVH
Bce-HPP   118  VASLHDFTKAIEEAKERWSKRGVTLKLGIEADYFIGGEQQLQSLLALG--DFDYVIGSVH
Bwe-HPP   118  VTSIYDFTKAIEEAKERWSKRGITLKLGIEADYFIEGEQELKGLLALG--DFDYVIGSVH
Mca-HPP   117  THKMDDFVTFINSQKEKWDKAGVELKLGIEADYFIGGEEELKSLLAPY--EFDYIIGSVH
Tth-HPP   51   MRLEALPFYLLALERVRERAQDLYVGIGLEADFHPGTEGFLAQLLRRY--PFDYVIGSVH
Bsu-HPP   60   AQASLERYIHEISGLKKEYRGQLSIRTGLEVDYIAEFEDEITLFLDTYGPYLDDSILSVH
                                     : :  * *                           .**

                                                                       ▼
Lla-HPP   110  EIGDIE-----------VYDG-TEFYLQKTKEEAQREYLLACLDVVQNFENYNSFGHLDY
Sb-PDE    161  YS--------------TFKQTGGQLRVDWNRTSADEKDLEARFREAKRQGAICQWNHPTI
Bce-HPP   176  FLNGWG-----------FDNLISACGSIEAVYEQYYRSIYSSIVASLGVYKPKRVGHITL
Bwe-HPP   176  FIDGWG-----------FDNPDTKEYFKEHDLYALYDTFFKTVECAVRSELFDIIAHLDN
Mca-HPP   175  FNHGWG-----------FDNPDTKEYFGTHELHTLYHTFFATVESAVRSELFDIIAHLDN
Tth-HPP   109  YLGAWP-----------LDHPELENKFNEYDLVKLYTDHFNTVIKAAESGIFSFIAHLDN
Bsu-HPP   120  FLRTDSSYLCLDYDEHTFKEPDHQEEYAWRDLKEVFRAYFQEVEKAARSGLFHAIGHLDL
                                . .                      .             *

Lla-HPP   158  VARYGPYTDKSIKFAENREILFEILRALASKEKALEINT-RLFDDPKTEQFYSDLLINFK
Sb-PDE    207  PSSSPG-------EAVVTPFLERMFSEGLLSGIEVAQSD-----------FIHPSAMDVA
Bce-HPP   225  IKVFN----YRLDENEQISYYKKIARALVETNTATEINAGLYYRYPVREMCPSPLYLQVL
Bwe-HPP   225  IKVFN----YRLDENEQLSYYKEIARALVETNTATEINAGLYYRYPVREMCPSPLYLQVL
Mca-HPP   224  LKVFN----YRPEEALLIPLYEQVAEALAKNDVATEVNVGLKYRYPVKEQCPSERFIKVL
Tth-HPP   158  PKKFG----HRLPEEALLELAEPALRAVAEAGLFLDVNT-AGLRRPAKEVYPAPALLRRA
Bsu-HPP   280  VQKFIK-LFPYSMSEHIRGLVSLCLNAIEENGMELDFNTSGLRKTYAGGIYIEDWMLNEA
                                                    .                  :

                          ▼ ▼
Lla-HPP   217  RLGGKFITLGTDSHIAKRDWLSIHKARTLIKKAGFHELATFSGMKIDKNKKSIKEKLAAA
Sb-PDE    249  LKYNLYIAATTDAHLGTALEQEAAGMDHRVATLFLTQGEDEAAFKDALEKRRTVGLFRD-
Bce-HPP   281  AKHEVPITLSSDAHYPNDLGKYVEENIKTLRNHDIAHLATFT--KRVRTMRLLEEVTISK
Bwe-HPP   281  AKHGVPITLSSDAHYPNDLGKYVEENIKTLRNHDISHIATFT--KRVRTMRLLEEEVIIS
Mca-HPP   280  SEYDVKFTTSSDSHFPHDIGIYNDEIRNLLKRNGVKSIVTFS--KMKREEKDIKSIIH--
Tth-HPP   213  RELGIGLVLGSDAHRPEEVGFAFPEVQALLAGLGFREAYYFV--EGSPVAYPLSRAS---
Bsu-HPP   239  KQKKIPLVFGSDAHQAGDVGYAYEAFLERC------------------------------
                     :.  :*:*

Lla-HPP   277  LE
Sb-PDE         --
Bce-HPP   339  --
Bwe-HPP   339  K-
Mca-HPP        --
Tth-HPP        --
Bsu-HPP        --
```

Figure S2 Amino acid sequence alignment of PHP domain of *Sb*-PDE (Ile55-Asp307) with L-histidinol phosphate phosphatase (HPP) from various organisms. *Lla*,

Lactococcus lactis subsp. *lactis* Il1403 (accession no. NP_267372); *Bce*, *Bacillus cereus* (WP_000861821); *Bwe*, *B. weihenstephanensis* (WP_078175627); *Mca*, *Macrococcus caseolyticus* (WP_012656252); *Tth*, *T. thermophilus* (WP_011227864); and *Bsu, B. subtilis* (WP_021480315). Identical residues and amino acid substitutions with low and high similarities are indicated by asterisks, and dots and double dots, respectively. Histidine, glutamate and aspartate residues which are metal coordination residues of L-histidinol phosphate phosphatase from *Lactococcus lactis* subsp. *lactis* Il1403 are indicated by triangles. Putative metal coordination residues of *Sb*-PDE are shown in reverse letters.
